# Increasing Role of Maternal Immune Activation in Neurodevelopmental Disorders

**DOI:** 10.3389/fnbeh.2018.00230

**Published:** 2018-10-05

**Authors:** Julie Boulanger-Bertolus, Carlo Pancaro, George A. Mashour

**Affiliations:** Center for Consciousness Science, Department of Anesthesiology, University of Michigan, Ann Arbor, MI, United States

**Keywords:** maternal immune activation, pregnancy, neonatal brain, neurodevelopmental disorders, autism spectrum disorder, schizophrenia, interleukin-6

## Abstract

The earliest stages of development are critically sensitive to environmental insults. An unfortunately timed stress on the developing brain can have dramatic consequences for the neurodevelopment and future mental health of the individual. In particular, infection of the mother during pregnancy has been correlated with increased risk of psychiatric and neurodevelopmental disorders. Evidence suggests that maternal immune activation, independently from the infection itself, can be responsible for the outcome in the offspring. This recognition has resulted in expanding study designs from epidemiologic correlations to the search for a causal relationship between activation of the maternal immune system and cognitive consequences for the offspring. However, this causality analysis remained limited in humans until recent work that longitudinally linked specific markers of maternal inflammation during pregnancy with alterations of the newborn brain and cognitive development of toddlers. This focused narrative review compares and discusses the results of these recent studies and places them into the broader landscape of maternal immune activation literature. New data point, in particular, to the association between the levels of interleukin 6 (IL-6) and modifications of the offspring’s salience network and subsequent cognitive impairments. This article further emphasizes the need to carefully control for potential confounders in studying the effects of maternal immune activation on the neonatal brain as well as the under-investigated consequences of intra-partum fever on offspring neurodevelopment.

Schizophrenia, autism spectrum disorder and cerebral palsy are disorders that can dramatically impact the quality of life of the afflicted individuals. Although their etiology is multifactorial and can involve both intrinsic and extrinsic factors, the likelihood to develop one of these conditions is suggested to be greatly increased by disruptions in the *in utero* environment (Antonelli, 2015). In particular, maternal inflammation during pregnancy is posited to play a strong role in their pathogenesis (Knuesel et al., 2014). Maternal immune activation can be strictly defined as measured levels of inflammatory markers exceeding normal range or more broadly defined as levels of these markers in the higher normal range. Infection, psychosocial stress, maternal psychopathology and high body mass index are common conditions associated with inflammatory states during pregnancy that, in turn, have been associated with increased rates of impaired neurodevelopment leading to mental disorders (Knuesel et al., 2014). These epidemiological observations have been reverse-translated in preclinical studies of rodents and non-human primates (Boksa, 2010; Bauman et al., 2014), suggesting that activating the maternal immune system, independently from the particular cause of that activation, can be responsible for the outcome in the offspring (Figure 1). These observations further established a causal link between maternal immune activation during pregnancy and changes in the offspring that mimic the symptoms of psychopathologies such as autism spectrum disorder or schizophrenia. A critically-timed imbalance in the expression of inflammation-related factors such as interleukin 6 (IL-6), IL-1α, IL-10, tumor necrosis factor α (TNFα), C-reactive protein (CRP) or the complement system has been suggested to play a role (Smith et al., 2007; Meyer et al., 2008; Boksa, 2010; Girard et al., 2010; Canetta et al., 2014; Coulthard et al., 2018). For example, in a model of mid-pregnancy viral infection, blocking the induced increase of IL-6 levels has been shown to prevent the negative consequences of the infection for the offspring, while on the other hand injections of IL-6 during pregnancy trigger long-lasting changes in the progeny behavior (Smith et al., 2007). Importantly, such an effect was not demonstrated for IL-1α, TNFα, or interferon γ (Smith et al., 2007), suggesting a key role for IL-6 in mediating the neurobehavioral consequences of infection for the offspring. Therefore, the prenatal immune environment can influence the earliest stages of brain ontogeny, possibly promoting the later development of disorders across multiple scales, from immediate disturbance of the neuronal development (Boulanger, 2009; Bilbo and Schwarz, 2012; Coulthard et al., 2018), to epigenetic alterations priming later susceptibility to a “second hit” (Estes and McAllister, 2016). However, human studies associating prenatal concentration of those factors, offspring brain development alteration and behavioral outcomes are still limited and would be crucial to better understand the consequences of maternal immune activation on the developing brain.

**Figure 1:**
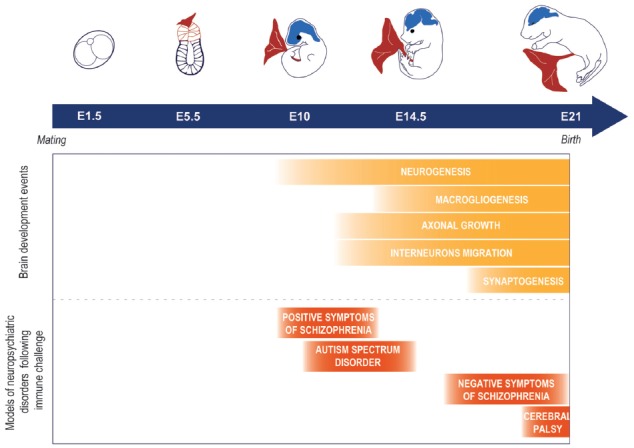
Example of models of neuropsychiatric disorders observed as a consequence of a prenatal immune challenge at different time points of the brain development in rodents. It is important to note that this is a simplification of the phenomenon and that other factors can play a role such as the nature of the infectious agent. For further information on the importance of the infectious agent and timing of the infection, see Fortier et al. (2007); Meyer et al. (2007); Careaga et al. (2017).

Recent work has, in part, addressed this gap in knowledge (Graham et al., 2018; Rasmussen et al., 2018; Rudolph et al., 2018; Spann et al., 2018). These studies had a similar longitudinal design by measuring the levels of inflammatory markers during pregnancy, followed by a correlation of those markers to network development using functional magnetic resonance imaging of the brain (Figure 2). Patterns of functional connectivity, the statistical co-variation between signals in different areas of the brain, was used as the surrogate of normal or abnormal development in large-scale brain networks (Graham et al., 2018; Rudolph et al., 2018; Spann et al., 2018). In some cases, structural connectivity, assessed by diffusion tensor imaging, provided additional insight into the physical neuronal architecture that forms the substrate for functional connectivity (Graham et al., 2018; Rasmussen et al., 2018). The studies further investigated the behavioral outcomes associated with higher concentrations of inflammatory markers by correlating their levels to the cognitive development of children between 12 and 24 months of age (Graham et al., 2018; Rasmussen et al., 2018; Rudolph et al., 2018; Spann et al., 2018; Figure 2). These longitudinal studies, from *in utero* to toddler age, allow the direct association of prenatal environment with the newborn’s brain development and later behavioral alterations. Adopting such an experimental design has the advantage of more carefully controlling for potentially confounding factors associated with the postnatal environment, allowing a more direct attribution of the observed effects to prenatal influences.

**Figure 2:**
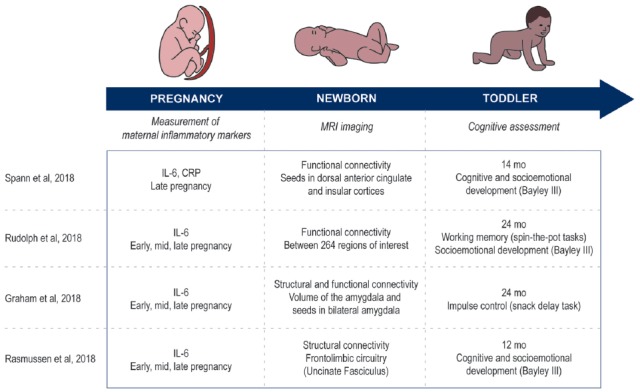
Comparison of the methods used in the articles reviewed. Study design in all four articles are longitudinal and measure three aspects: inflammatory markers during pregnancy, imaging the newborn’s brain and cognitive development of the toddler.

In an adolescent population at higher risk for maternal immune activation due to psychosocial stress, Spann et al. (2018) measured the levels of the inflammatory markers IL-6 and CRP during late pregnancy (Figure 2). Shortly after birth (age at scanning—mean ± SD: 2.6 weeks ± 0.53), they scanned the sleeping newborns and looked at relevant structures of the salience network by performing a whole-brain seed connectivity analysis from the dorsal anterior cingulate cortex and the left and right insula. Their results suggest correlations between the concentrations of IL-6 and CRP and the functional connectivity patterns of regions of the salience network with cortical and subcortical structures at birth. To give some examples of how immune activation can increase network connectivity, higher maternal IL-6 was associated with higher functional connectivity between the left insula and both the medial prefrontal cortex and occipital gyrus. Higher maternal CRP during pregnancy was associated with greater connectivity between the left insula and right temporoparietal junction, the right insula and basal ganglia, and the dorsal anterior cingulate and cuneus, temporoparietal junction and extrastriate cortex. Many of these areas are of potential relevance to neurodevelopmental or psychiatric disorders. For example, the temporoparietal junction is thought to be critical for normal consciousness (Koch et al., 2016), multisensory integration (Ronchi et al., 2018) and attributing awareness to others (Kelly et al., 2014). The insula is thought to be critical for interoception and has been posited to be the site of the “sentient self” (Craig, 2009).

A broader context for the results of Spann et al. (2018) is established by comparing them to three studies that investigated the same population of mother-child dyads at the University of California Irvine (UCI), correlating the levels of IL-6 during early, mid and late pregnancy to characteristics of the neonate’s brain (age at scanning—mean ± SD: 3.8 ± 1.8) measured when the infant was sleeping in the scanner (Graham et al., 2018; Rasmussen et al., 2018; Rudolph et al., 2018; Figure 2). Differently than Spann et al. (2018) the UCI studies measured the IL-6 levels of non-teenage mothers (mother’s age—mean ± SD: 28.2 ± 5.5 vs. 17.6 ± 3.4), a population less at risk for psychosocial stress. As was predicted, teenage pregnancy was associated with a tendency to higher IL-6 levels than observed in pregnancies later in life (Spann et al. (2018): 1.7 pg/ml ± 1.0, *n* = 36 vs. UCI studies: 1.2 pg/ml ± 1.3, *n* = 86; *t*_(120)_ = 1.87, *p* = 0.064). Nevertheless, the IL-6 levels were still correlated with modifications of the neonate brain in the UCI studies. More specifically, the average level of maternal IL-6 during pregnancy was associated with modifications of the: (1) volume of the right amygdala, and of the connectivity between bilateral amygdala and regions involved in sensory processing and integration, salience detection, and learning and memory, as measured using whole-brain analysis (Graham et al., 2018); (2) fronto-limbic microstructural maturation, as estimated from the fractional anisotropy of water diffusing in the brain (Rasmussen et al., 2018); and (3) functional connectivity within the salience network and between structures involving the subcortical network, the salience network and the dorsal attention network, as measured by pairwise correlations of activity within or between 264 regions of interest belonging to larger networks or communities (Rudolph et al., 2018). Taken together, these studies support the hypothesis that increased IL-6 levels are likely associated with modifications of the salience system as suggested by Spann et al. (2018). Faced with an abundance of incoming stimuli at the same time, the salience network has a central role in detecting and filtering those that are behaviorally relevant, considering parameters such as our past experience, our current homeostatic state, or our goals. Its atypical functioning has been increasingly implicated in neuropsychiatric disorders such as autism spectrum disorder and schizophrenia (Uddin, 2015). An association between the levels of IL-6 and altered neurodevelopment of the salience network in human neonates gives a first glimpse of the mechanisms underlying how infection during pregnancy may prime the brain for mental disorders.

The studies further investigated how levels of inflammatory markers and brain alterations were associated with the subsequent behavioral development of the offspring (Figure 2). At 24-months, Graham et al. (2018) showed through a snack delay task, where a child waited to retrieve a candy from a see-through cup, that a higher concentration of IL-6 during pregnancy was associated with lower impulse control. This association was mediated by a stronger amygdala-fusiform connectivity. Furthermore, Rudolph et al. (2018) showed that IL-6 levels throughout the pregnancy, but especially during the 3rd trimester, could predict working memory performance at 24 months. Working memory was measured by a spin-the-pot task, where children are asked to remember in which six of eight pots they hid stickers, and higher IL-6 levels was associated with a decreased score on the task. No association was found between IL-6 levels during pregnancy and offspring negative emotionality using the Social-Emotional scale of Bayley Scales for Infant and Toddler Development (Bayley-III) at 12 months, or the Infant Behavior Questionnaire at 24 months (Rasmussen et al., 2018; Rudolph et al., 2018). Interestingly, however, the studies found conflicting results when measuring the cognitive development of the offspring: while Spann et al. (2018) found a positive correlation between concentrations of inflammatory markers during pregnancy and cognitive development at 14 months using the Cognitive Scales of the Bayley-III, Rasmussen et al. (2018) found a negative correlation using the same scale at 12 months. Further analyses of these results showed that changes in fractional anisotropy during the first 12 months of life, a measure of changes in the fronto-limbic microstructural maturation, mediated the association between maternal IL-6 levels during pregnancy and cognitive development at 12 months (Rasmussen et al., 2018). On the other hand, Spann et al. (2018) found no mediating effect of the connectivity between the dorsal anterior cingulate and the medial prefrontal cortex on the association between maternal IL-6 or CRP levels and cognitive scores of the offspring. Overall, these results may seem hard to reconcile. However, there are several key methodological differences. For example, the network nodes in question were somewhat different and Spann et al. (2018) used functional connectivity as opposed to the structural connectivity measure applied by Rasmussen et al. (2018). This apparent contradiction could also be explained by the fact that Rasmussen et al. (2018) adjusted the scores for interindividual variation in the postnatal caregiving environment. The mothers studied by Spann et al. (2018) are adolescent pregnant women, a population at high risk of psychosocial stress from low socioeconomic status, poor social support and poor nutrition. Thus, it is likely that the postnatal environment differed significantly between the two studies, suggesting a strong interaction between the prenatal and postnatal environment that would be of pivotal role in influencing the developing brain. Furthermore, the teenage mothers studied by Spann et al. (2018) had a tendency to show higher IL-6 levels than the UCI population, which could also play a role here. The authors suggest that a positive correlation between inflammatory markers and cognitive development at 14 months could reflect an adaptive neurodevelopmental response (Spann et al., 2018). Such adaptation has been observed in the context of prenatal and early postnatal development and suggests that the developing brain is resilient when exposed to a mild stressor since it can develop protection against future stressors in response to it (Fujioka et al., 2001; Cannizzaro et al., 2006; Li et al., 2013). Another interpretation relates to a possible sex effect. Indeed, previous work has highlighted the influence of sex differences on the consequences of prenatal challenges (Carpenter et al., 2017). For example, Li et al. (2013) has shown that maternal exposure to stressful life events during pregnancy is associated with school achievement that is lower in girls but higher in boys. Similarly, high levels of IL-6 during pregnancy could increase the cognitive development scores at 1 year in boys but decrease them in girls, thus accounting for some of the difference in the results reported by Spann et al. (2018; 67% boys) vs. those described by Rasmussen et al. (2018; 56% boys). Therefore, multiple factors could be involved in the discrepancy between the studies’ results and would deserve further investigation.

In the four studies reviewed here, it is worthwhile noting that the reported IL-6 levels were not outside of the normal limits. Nonetheless, IL-6 levels were correlated to the offspring outcome, suggesting that even modest and subthreshold variations in maternal IL-6 are associated with altered neonatal functional connectivity and later cognitive function in humans. It therefore seems reasonable to hypothesize that even subtle changes in the intra-uterine milieu during critical time windows affect the offspring. For example, mild changes within physiologic levels in maternal hormone levels can modify gene expression profiles in the offspring (Miranda and Sousa, 2018). Studies in rodent models of maternal infection further support this observation, suggesting that even subclinical maternal infection can increase the likelihood of environmental and genetic risk factors for psychiatric disorders such as autism spectrum disorder and schizophrenia (Meyer, 2014). The mechanism by which the increase in IL-6 alters the neurodevelopment of the offspring remains poorly understood (Boksa, 2010). Rodent studies have demonstrated that maternal IL-6 can directly cross the placenta and reach the fetus during mid- but not late gestation (Dahlgren et al., 2006). Later in pregnancy, the placenta could play a key role in translating maternal immune activation to an inflammation of the placenta and fetal brain, as deleting IL-6 receptors in the placental trophoblasts of mice prevented negative outcome induced by prenatal infection (Wu et al., 2017). However, how this acute response to inflammation of the placenta and the fetus mediates long-term modification of the offspring’s brain and behavior warrants further investigation.

It is also important to note that Spann et al. (2018) found a correlation between IL-6 levels in pregnancy and the type of delivery, showing that C-sections, spontaneous vaginal deliveries and assisted vaginal deliveries were respectively associated with the highest, intermediate and lowest levels of IL-6 during pregnancy. Although unexplained at this point, this detail may be of importance, and controlling for the conditions of birth in similar studies may be crucial. Even in the case of healthy mothers, non-infectious maternal fevers is observed in up to 1 in 5 laboring mothers undergoing epidural analgesia (Sultan et al., 2016). Nulliparity is a risk factor for epidural-associated intrapartum fever (Segal, 2010), which may be of relevance when comparing teenage and adult populations. Importantly, such fever is associated with a significant increase in maternal and fetal IL-6 levels (Goetzl et al., 2002). Exposure to high levels of pro-inflammatory cytokines around the time of neonatal brain imaging is a potential confounding factor for which it is important to control. It could further have direct consequences on the neonatal brain, as an increasing number of epidemiological and preclinical studies suggest that intra-partum fever is associated with negative neonatal outcome such as encephalopathies or cerebral palsy (Segal, 2010; Seri et al., 2012; Segal et al., 2017). Furthermore, maternal fever at birth has been associated with increased long-term cognitive deficits in preterm infants (Dammann et al., 2003) and disorders such as schizophrenia and autism may also be related to intrapartum maternal fever (Segal, 2010). It is important to note that fever during labor was recently found to have benefited from the least amount of research advances in the last decade compared to a selection of other parturition-related topics (Lim et al., 2018). Thus, there is a critical need for more research in this area.

The articles briefly reviewed here (Graham et al., 2018; Rasmussen et al., 2018; Rudolph et al., 2018; Spann et al., 2018) represent a turning point in the study of prenatal exposure to maternal immune activation. The recent advances in our ability to image the developing brain allow a pivot from epidemiological and preclinical studies to direct observations of altered brain functioning associated with prenatal insults. Future work should address in greater depth the interaction between prenatal exposure to maternal immune activation, the consequences for the offspring’s neuronal and cognitive development, and the postnatal rearing environment and sex of the newborn. Comparison of the respective strengths of the four articles described here suggests that future studies would benefit from the simultaneous measure of several markers of inflammation performed at multiple time points, with extra care being taken to control for parturition-associated events and delivery mode, the postnatal environment and possible sex differences. Better understanding of how these factors interact would help the development of successful interventions for the prevention of neurodevelopmental disorders.

## Author Contributions

JB-B wrote the first draft of the manuscript. All authors contributed to manuscript revision, read and approved the submitted version.

## Conflict of Interest Statement

The authors declare that the research was conducted in the absence of any commercial or financial relationships that could be construed as a potential conflict of interest.
